# Increased cortical excitability to transcranial magnetic stimulation at the brain-tumor interface of *IDH1*-mutant gliomas

**DOI:** 10.1093/noajnl/vdag071

**Published:** 2026-03-15

**Authors:** Alexia Stark, Mykola Gorbachuk, Kathrin Machetanz, Maria Teresa Leao, Marina Liebsch, Sophie Wang, Jürgen Honegger, Marcos Tatagiba, Georgios Naros

**Affiliations:** Neurosurgical Clinic, Department of Neurosurgery and Neurotechnology, Eberhard Karls University, Tuebingen, Germany; Neurosurgical Clinic, Department of Neurosurgery and Neurotechnology, Eberhard Karls University, Tuebingen, Germany; Neurosurgical Clinic, Department of Neurosurgery and Neurotechnology, Eberhard Karls University, Tuebingen, Germany; Neurosurgical Clinic, Department of Neurosurgery and Neurotechnology, Eberhard Karls University, Tuebingen, Germany; Neurosurgical Clinic, Department of Neurosurgery and Neurotechnology, Eberhard Karls University, Tuebingen, Germany; Neurosurgical Clinic, Department of Neurosurgery and Neurotechnology, Eberhard Karls University, Tuebingen, Germany; Neurosurgical Clinic, Department of Neurosurgery and Neurotechnology, Eberhard Karls University, Tuebingen, Germany; Neurosurgical Clinic, Department of Neurosurgery and Neurotechnology, Eberhard Karls University, Tuebingen, Germany; Neurosurgical Clinic, Department of Neurosurgery and Neurotechnology, Eberhard Karls University, Tuebingen, Germany

**Keywords:** brain-tumor interface, epileptogenesis, glioma, isocitrate dehydrogenase, transcranial magnetic stimulation

## Abstract

**Background:**

There is increasing interest in the glioma-to-neuro communication at the brain-tumor interface (BTI). In vitro studies indicate that gliomas with a mutation of the isocitrate dehydrogenase (*IDH*) increase neuronal excitability of the peritumoral cortex, contributing to epileptogenesis in these patients. However, in vivo evidence is missing. This study evaluates the electric characteristics of the BTI relative to the *IDH* mutation status.

**Methods:**

To investigate peritumoral cortical excitability (CE), we applied 5258 pulses of transcranial magnetic stimulation (TMS) at the BTI of *IDH*-mutant (*IDH-mt*) and *IDH*-wildtype (*IDH-wt*) glial tumors in 39 patients. Cortical excitability was assessed by the resting motor threshold (RMT) and the synchronized electromyographic (EMG) activity (ie, event-related spectral perturbation, ERSP) after TMS. The ERSP values were related to the *IDH* status, tumor grading, antiepileptic drug (AED) intake, and the spatial relationship to the tumor borders.

**Results:**

Within our sample, there was no significant group difference in RMT. The TMS to the BTI triggered an EMG synchronization decreasing linearly with the distance to the functional hotspot. In contrast, *IDH-mt* gliomas demonstrated an increased cortical output of the peritumoral brain tissue compared with *IDH-wt* gliomas. This effect was not attributable to AED intake or other histological and molecular characteristics. Notably, cortical hyperexcitability was detectable well beyond the tumor border.

**Conclusions:**

This study provides in vivo evidence of cortical hyperexcitability at the BTI of *IDH-mt* gliomas. The data demonstrate how molecular glioma characteristics affect peritumoral neuronal circuits. Modulating interactions at the BTI might pave the way for novel therapies.

Key Points

- Glioma-to-neuron interactions define peritumoral excitability and epileptogenesis in glioma.
- The study provides in vivo data for peritumoral hyperexcitability in *IDH*-mutant gliomas.
- Understanding the brain-tumor interface might pave the way for novel therapies.

Importance of the Study

In vitro studies indicate that bidirectional glioma-to-neuro communication at the brain-tumor interface affects tumor cell growth and migration as well as the peritumoral neuronal activity. This peritumoral hyperexcitability is suggested to contribute to epileptogenesis in glioma with a mutation of the isocitrate dehydrogenase (*IDH*). However, in vivo evidence is missing. By means of transcranial magnetic stimulation (TMS), this study provides first in vivo data for peritumoral hyperexcitability in *IDH*-mutant gliomas compared to *IDH*-wildtype gliomas. This effect was not attributable to antiepileptic drug intake or other histomolecular characteristics and was detectable well beyond the tumor border. The data demonstrate how molecular glioma characteristics affect peritumoral neuronal circuits at the brain-tumor interface. Modulating the interactions at the brain-tumor interface might pave the way for novel glioma therapies.

For many years, glioma tissue has been considered unresponsive to external stimulation with no direct functional interactions to the surrounding tissue.^1^ In vitro studies, however, have identified an integration of glioma cells into adjacent neural circuits affecting the excitability of the peritumoral cortex^2–5^ and contributing to the epileptogenesis in these patients. It was not until recently that an association of distinct glioma genetic profiles and cortical hyperexcitability was established.^5^^,^^6^ Furthermore, interactions at the brain-tumor interface (BTI) are affecting tumor progression^7^ and oncological outcome.^8^ Understanding the mechanisms at the BTI might pave the way for new approaches to glioma therapy.^8–10^ However, direct evidence for functional interactions at the BTI from in vivo human data is sparse.^2^^,^^3^^,^^5^

Gliomas with mutations of the metabolic enzyme “isocitrate dehydrogenase” (*IDH-mt*) are known to have a better clinical prognosis than those with its wildtype (*IDH-wt*).^11^ However, the *IDH-mt* status is an independent risk factor for epileptogenesis in glioma patients.^11^^,^^12^ A recent in vitro study provides evidence that *IDH-mt* gliomas increase neuronal excitability in the surrounding tissue by metabolic interaction.^4^ Interestingly, peritumoral hyperexcitability was no longer evident following tumor resection.^5^

In vivo, cortical excitability is routinely probed by transcranial magnetic stimulation (TMS) to the motor cortex.^13^^,^^14^ The external magnetic input leads to a corticospinal output detectable as synchronized electromyographic (EMG) activity, the so-called motor-evoked potential (MEP).^13^^,^^14^ Fluctuations of cortical excitability are known to affect the corticospinal output (ie, the MEP amplitude) after TMS.^15^ Cortical excitability in response to TMS is thought to be regulated by complex synaptic interactions between excitatory and inhibitory interneurons.^16^ An imbalance of, presumably, similar excitatory and inhibitory circuits mediates the cortical hyperexcitability associated with epileptogenesis.^16^ Transcranial magnetic stimulation has been used to track cortical excitability in epilepsy patients.^17^^,^^18^ However, there is limited information about the effect of the *IDH* mutation status on peritumor excitability.^19–22^

The present study evaluates the output gain of peritumoral brain tissue after TMS in 39 *IDH-mt* and *IDH-wt* glioma patients, controlling for other histological and molecular tumor characteristics, antiepileptic drug intake (AED), and the exact spatial relationship to the tumor borders and the functional hotspot.

## Methods

### Patients

This prospective study recruited 39 patients with suspected glial brain tumors in motor eloquent areas (47.1 ± 15.1 [18-75] years, 14 female) undergoing a navigated TMS examination prior to elective tumor resection between 2010 and 2015. The inclusion criteria were (1) age >18 years, (2) no contraindication against TMS, (3) histopathological diagnosis of a glioma, and (4) no clinically detectable motor deficits. Gliomas were classified as low-grade (LGG, World Health Organization [WHO] grade I and II) or high-grade (HGG, WHO grade III and IV) by a board-certified neuropathologist according to the WHO classification of 2016.^23 ^The Medical Research Council Scale (MRCS) and the Grooved Pegboard Test (GPT) were used to determine patients’ motor status and dexterity of the contralateral upper limb, respectively. Routinely, glioma classification integrates microscopical features (eg, anaplasia signs such as necrosis [NEC] and microvascular proliferation [MVP]) as well as histological and molecular markers, for example: missense mutations in codon 132 of the isocitrate dehydrogenase enzyme (*IDH1*), loss of nuclear expression of the alpha-thalassemia/intellectual disability syndrome X-linked gene (*ATRX*), methylation of the O6-methylguanine-DNA methyltransferase (*MGMT*), Mindbomb Homolog 1 (*MIB*) antibody labeling, and loss of heterozygosity of the 1p and 19q chromosomal arms (*1p/19q LOH*). The *IDH1* status was determined by a pyrosequencing assay separating patients into *IDH-mt* and *IDH-wt.*^24^ Details of the clinical and demographic characteristics are depicted in Table 1. This study was approved by the local ethics committee of the Medical Faculty of the Eberhard Karls University Tuebingen. All participants gave written informed consent.

**Table 1.** Patients’ clinical and imaging characteristics

	*IDH-mt*	*IDH-wt*	
	*n = 19*	*n = 20*	
**Age**	43.0 ± 13.1	50.9 ± 16.2	*H = 3.66; P = .056; Kruskal*
**Gender (f:m)**	6:13	8:12	*χ^2^ = 0.30, P = .416; Fisher*
**Height (cm)**	177 ± 10	174 ± 9	*H = 0.22; P = .640; Kruskal*
**Weight (kg)**	83.7 ± 23.5	79.0 ± 11.8	*H = 0.10; P = .757; Kruskal*
**Diagnosis**			
* HGG:LGG*	9:10	15:5	*χ^2^ = 3.14, P = .074; Fisher*
** HGG**	9	15	
* ATRX+*	7	11	*χ^2^ = 0.59, P = .603; Fisher*
* MGMT+*	13	8	*χ^2^ = 0.53, P = .397; Fisher*
* MIB (%)*	8 ± 4	22 ± 12	***H = 8.67; P = .003; Kruskal***
* NEC/MVP+*	2	13	***χ^2^ = 9.97, P = .003; Fisher***
** LGG**	10	5	
* ATRX+*	8	5	*χ^2^ = 1.15, P = .429; Fisher*
* 1p/19q LOH*	7	0	***χ^2^ = 6.56, P = .019; Fisher***
* MIB (%)*	5 ± 4	3 ± 2	*H = 0.97; P = .756; Kruskal*
* NEC/MVP+*	–	–	*–*
** WHO**			
* WHO I*	0	1	*χ^2^ = 0.98, P = 1.000; Fisher*
* WHO II*	11	3	***χ^2^ = 7.27, P = .007; Fisher***
* WHO III*	8	2	***χ^2^ = 4.33, P = .037; Fisher***
* WHO IV*	0	14	***χ^2^ = 20.75, P < .001; Fisher***
**AED**			
Yes:No	12:7	16:4	*χ^2^ = 1.37, P = .209; Fisher*
**Tumor size (cm³)**	34.8 ± 23.6	39.1 ± 43.4	*H = 1.39; P = .238; Kruskal*

Abbreviations: *1p/19qLOH*, loss of heterozygosity of the 1p and 19q chromosomal arms; *AED*, antiepileptic drugs; *ATRX*, alpha-thalassemia/intellectual disability syndrome X-linked gene; *HGG*, high-grade glioma; *IDH-mt*, mutation of isocitrate dehydrogenase; *IDH-wt*, wildtype of isocitrate dehydrogenase; *LGG*, low-grade glioma; *MGMT*, O6-methylguanine-DNA methyltransferase; *MIB*, Mindbomb Homolog 1; *MVD*, microvascular proliferation; *NEC*, necrosis. Statistical significance is indicated in bold.

### Magnetic Resonance Imaging

**Figure 1.**
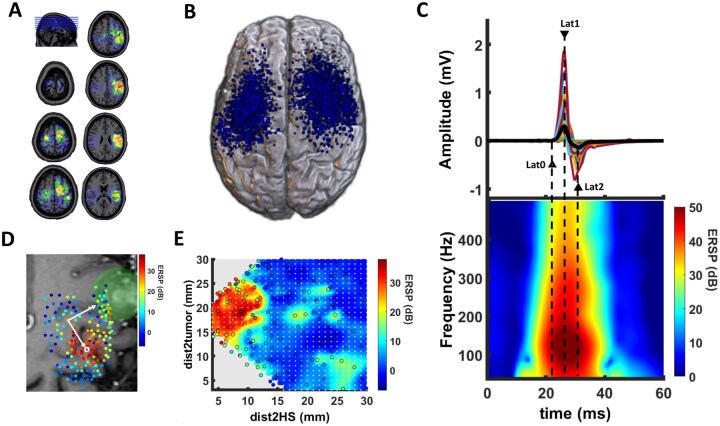
Study design. (A) Overview of patients’ glioma localizations in common space indicates a spatial relation to motor-eloquent areas. (B) In total, we applied 5258 magnetic pulses to the brain tissue in the vicinity of the gliomas. The figure (blue dots) displays the localization of the 2042 stimulation sites (SS) resulting in motor-evoked potentials (MEP) with a peak-to-peak amplitude ≥ 20 µV (ie, MEP+ trials). (C) The MEP time-series demonstrated a high inter-subject variability (*upper row*). In the time-frequency domain, MEP are represented by a positive event-related spectral perturbation ERSP (ie, synchronization) of the electromyographic (EMG) signal for 20-40 ms after TMS. Triangles (▲) indicate the mean values for Lat0 (onset latency), Lat1 (latency of positive peak) and Lat2 (latency of negative peak). (D) TMS provides a functional mapping of the peritumoral brain tissue. For each SS, we determined the closest distance-to-tumor (dist2tum) and the distance-to-hotspot (dist2HS) (yellow arrows). *Green*: glioma; *yellow cross*: functional hotspot; *white*: MEP- stimulation sites; *colored dots*: MEP+ stimulation sites; *red*: SS with dist2tum ≤15 mm; *blue*: SS with dist2tum >15 mm; *black*: SS with dist2HS ≤ 10 mm. (E) To explore the spatial relationship between the corticospinal output gain, the tumor and the functional hotspot, we rearranged stimulation sites (white dots) according to dist2HS (*x*-axis) and dist2tumor (*y*-axis) in a 2-dimensional coordinate system (ie, spatial map).

All patients received anatomical magnetic resonance imaging (MRI) using a 1.5 T MRI unit (Skyra/Prisma-fit/Aera, Siemens Healthineers, Erlangen, Germany) with an 8-channel head coil (T2 and contrast-enhanced T1-weighted echo sequence). The anatomical MRI data set was imported to the TMS system (Nexstim Eximia, version 3.2.2, Helsinki, Finland) for cortical mapping. An experienced neurosurgeon manually delineated each tumor on MRI using the Mricro software (https://people.cas.sc.edu/rorden/mricro/mricro.html). The individual tumor volume was noted and the tumor mask was saved for further analysis. Subsequently, the individual lesion maps were spatially normalized using the normalization parameters obtained from the individual co-registered T1 scan by the SPM8 software^25^ (Figure 1A).

### Transcranial Magnetic Stimulation

The cortical mapping procedure has been described previously in detail.^26^^,^^27^ Prior to the mapping, patients’ anatomical T1-weighted MRI were co-registered to the patient’s head with a registration error of <2 mm. nTMS mapping was performed with a biphasic figure-8 coil (eXimia, Nexstim, Helsinki, Finland). After determining the “hotspot” yielding the largest MEP from the contralateral first dorsal interosseous muscle (FDI), the resting motor threshold (RMT) was obtained, defined as the minimum stimulus intensity that resulted in an MEP > 50 µV in at least 5/10 trials. Coordinates of the hotspot and the RMT were saved for further analysis. The orientation of the electric field (EF), calculated based on the individual MRI of each subject by the eXimia software, was kept perpendicular to the mapped sulcus. The cortex was mapped with 110% RMT starting at the primary motor cortex and then extending around this spot to cover the primary motor cortex, somatosensory cortex and premotor cortex. In total, we applied 5258 magnetic pulses. On average, 134.5 ± 74.6 [22-336] stimuli were applied per patient and map. Of 5258 stimulations, 2042 (39%) evoked an MEP with a peak-to-peak amplitude ≥20 µV (MEP+ trials), which were selected for further analysis (Figure 1B). Coordinates and the induced EF strength were automatically saved by the eXimia software for each stimulation site (SS). The Matlab function “*convhull.m*” was used to calculate the convex hull enveloping the x and y coordinates of the SS. The area (in cm^2^) of the convex hull represents the extent of the mapping area (MAParea_tot). The analysis was repeated for MEP+ trials, resulting in the area of the cortical FDI representation (MAParea_pos). Custom-written scripts based on MATLAB (MathWorks Ltd, USA, R2017a) were used to automatically calculate the minimum distance from each SS to the borders of the tumor mask (*dist2tum*) and to the hotspot (*dist2HS*). Additionally, we calculated the minimum distance between the hotspot and the tumor mask (*distHS2tum*) (Figure 1C). We rearranged stimulation sites according to dist2HS (*x*-axis) and dist2tum (*y*-axis) in a 2-dimensional coordinate system (ie, spatial map). Individual SS coordinates were harmonized by triangulation-based cubic interpolation (Matlab “griddata.m” function) resulting in a 1 × 1 mm resolution (ie, 0 ≤ dist2HS ≤ 30 mm and 0 ≤ dist2tum ≤ 30 mm) of the spatial maps (Figure 1D). For group analysis, we averaged the spatial maps across subjects.

### Data Analysis

During TMS mapping, EMG of the contralesional FDI was recorded with the eXimia EMG amplifier system (3 kHz sampling rate, band-pass filter of 10-500 Hz) using Ag/AgCl wet gel surface electrodes (AmbuNeuroline 720, Ambu GmbH, Germany). Data analysis was performed with custom-written scripts based on MATLAB and its open-source toolboxes EEGlab^28^ and Fieldtrip.^29^ The EMG data were segmented into epochs from −100 to +100 ms relative to the TMS pulse. There was no further data processing except for linear detrending of the epochs. The TMS trials were classified as MEP+ trials depending on an MEP amplitude (≥20 µV). Trials with artifacts or EMG pre-stimulus activation were automatically removed from analysis. A Matlab-based custom-written script was used to automatically detect several time series characteristics of the MEP: Amp (ie, peak-to-peak amplitude), Lat0 (ie, MEP onset latency), Lat1 (ie, latency of the maximum positive deflection of the MEP) and Lat2 (ie, latency of the minimum negative deflection of the MEP). Additionally, MEP were transferred to the frequency domain, an alternative description of MEP which has been shown to be more reliable and sensitive to detect intra-subject and intersubject effects on corticospinal output in glioma patients.^26^^,^^27^^,^^30^ The time-frequency analysis of the MEP was performed on the basis of a Morlet wavelet approach with a fixed wavelet length of 40 ms (as implemented by the *newtimef* function of the EEGlab toolbox).^28^ This approach resulted in a spectral resolution of 1 Hz (37-500 Hz) and a temporal resolution of 0.333 ms (−79.333 to 79.333 ms relative to the TMS pulse). Event-related spectral perturbations (ERSPs) were calculated (in dB) and trial-wise normalized to the baseline spectrum (−79.3 to 0 ms relative to the TMS pulse)^31^ (Figure 1E). The ERSP values of different subsets of SS were averaged to achieve ERSP_all_ (across all MEP+ trials), ERSP_HS_ (SS at the HS, ie, dist2HS ≤ 10 mm) and ERSP_NHS_ (SS distant to the HS, ie, dist2HS > 10 mm), ERSP_BTI_ (SS at the BTI, ie, dist2HS > 10 mm and 11 mm ≤ dist2tum ≤ 15 mm). To avoid pseudo-replication, ERSP maps were averaged at the patient level. For each subject, one ERSP map per condition (ERSPall, ERSP_HS_, ERSP_NHS_, ERSP_BTI_) was generated, and all statistical comparisons of time-frequency data were performed across patients (n = 39). To quantify excitability at the BTI, we defined a BTI zone comprising stimulation sites located >10 mm from the functional hotspot and 11 to 15 mm from the tumor border. For these sites, a cluster-averaged ERSP value was computed (ERSP_BTI_)

### Statistics

Statistical evaluation was performed using SPSS (IBM SPSS Statistics for Windows, Version 25.0, Armonk, NY: IBM Corp.) and custom-written Matlab scripts including the FieldTrip and Matlab statistics toolbox. Group effects on clinical (eg, age, gender, diagnosis), imaging (eg, tumor volume), and electrophysiological characteristics (RMT, no. of trials, MEP amplitudes and latencies) were evaluated by nonparametric Kruskal-Wallis and χ^2^ tests, when applicable.

A cluster-based permutation test implemented in the Fieldtrip toolbox (*ft_freqstatistics*.m) was used to explore the time-frequency MEP representation when comparing 2 different cohorts (ie, *IDH-mt* vs *IDH-wt*, AED+ vs AED−, LGG vs HGG). In general, time-frequency data are characterized by high spectro-temporal dimensionality. After transforming into the time-frequency domain, the MEP signal is represented by a large number of (time, frequency)-samples. A pairwise statistical comparison would result in a major multiple comparisons problem (MCP). The cluster-based test statistics can help to reduce the dimensionality of the data and to solve the MCP by determining significant spectro-temporal clusters. Each (time, frequency)-sample was compared across the different conditions by an unpaired 2-tailed *t* test (*ft_statfun_indepsamplesT.m*). *t* values were thresholded at the 2.5-th and the 97.5-th quantiles for a 2-sided test. Selected samples were clustered in connected sets according to temporal and spectral adjacency. Cluster-level statistics were then calculated by taking the sum of the *t* values within every cluster, and the resultant maximum summed *t* values were used to compute the statistical comparisons. The significance probability was calculated using a Monte-Carlo method (with 1000 permutations).^32^ By randomizing the data, the reference distribution of the maximum of summed cluster *t* values was acquired. Clusters from the original data were considered to be significant if their summed cluster *t* values were below or above the 2.5-th and the 97.5-th quantiles of the reference distribution (representing a 2-sided test with an alpha level of 5%). For each stimulation site (SS), a single ERSP value was computed as the mean ERSP amplitude within the significant time-frequency cluster (37-200 Hz, 20-40 ms). In a second step, we sought to confirm the ERSP results of the cluster-based analysis by univariate Kruskal-Wallis analysis of variance and a multivariate linear regression including the *IDH* status, AED intake, tumor GRADE, and EF as independent variables. Results are shown as mean ± standard deviation (SD).

## Results

### Patient Characteristics

The present study enrolled 39 patients with motor-eloquent glial brain tumor but no apparent motor deficits. Clinical details are summarized in Table 1. In 19/39 (49%) patients, a mutation of the isocitrate dehydrogenase 1 enzyme was detected (*IDH-mt*). *IDH-mt* gliomas presented a smaller proliferation rate and less frequent microscopic signs of anaplasia (ie, necrosis, microvascular proliferation) than *IDH-wt* glioma. In summary, 24/39 (62%) were graded by histological and molecular means as HGG (WHO grade III and IV). The *IDH* status was equally distributed in the HGG and LGG. Within the *IDH-mt* group, there were 7 patients with LOH+ tumor representing oligodendroglioma. Demographic parameters were equivalent in both *IDH* groups. Notably, *IDH-mt* and *IDH-wt* patients did not differ in regard to presurgical seizures or the need for AED intake. There was no difference in tumor volume between *IDH-mt* and *IDH-wt* patients. All tumors were located in the vicinity of motor-eloquent brain regions (Figure 1A).

### Transcranial Magnetic Stimulation

Patients underwent TMS motor mapping in an identical matter. Detailed results are summarized in Table 2. In total, we applied 5258 magnetic pulses to motor-eloquent brain tissue in the vicinity of the tumor. Of 5258 stimulations, 2042 (39%) evoked an MEP (ie, MEP+ trials, Figure 1B). There were no group differences between *IDH-mt* and *IDH-wt* patients in regard to the brain area mapped by TMS (MAParea_tot) or the cortical representation (MAParea_pos) of the contralateral upper limb (Table 2). We determined the distance-to-tumor (dist2tum) and the distance-to-hotspot (dist2HS) for each SS (Figure 1C) and rearranged SS according to dist2HS and dist2tum in a 2-dimensional spatial map for intersubject comparison (Figure 1E). The average distance between the MEP+ stimulation sites and the tumor border (*dist2tum*) was comparable in both groups. Notably, 50% of all MEP+ stimulation sites (ie, interquartile range, IQR) were located within 14.9 and 16.0 mm of the tumor borders, respectively (Table 2). In contrast, the mean distance between the functional hotspot and the tumor border (*distHS2tum*) was approximately 11.5 mm (Table 2).

**Table 2.** Patients’ electrophysiological characteristics

	*IDH-mt*	*IDH-wt*	*p-value (Kruskal-Wallis)*
**RMT (%)**	*42.6 ± 7.6*	*39.0 ± 9.8*	*H = 2.85; P = .091*
**EF_HS_ (V/m)**	*63.6 ± 21.9*	*64.8 ± 26.2*	*H = 0.03; P = .955*
**no. of trials**	*127 ± 72*	*142 ± 90*	*H = 0.24; P = .623*
**MEP+ trials**	*52 ± 24*	*53 ± 30*	*H = 0.07; P = .789*
**MEParea_tot (cm²)**	*12.2 ± 5.0*	*11.5 ± 7.7*	*H = 0.29; P = .593*
**MEParea_pos (cm²)**	*6.4 ± 3.5*	*5.1 ± 3.7*	*H = 1.97; P = .160*
**dist2tum [IQR] (mm)**	*9.8 ± 8.6 [14.9]*	*10.8 ± 10.0 [16.0]*	*H = 1.36; P = .244*
**dist2HS [IQR] (mm)**	*11.9 ± 7.2 [8.7]*	*12.1 ± 7.9 [9.1]*	*H = 0.12; P = .731*
**distHS2tum [IQR] (mm)**	*11.5 ± 7.6 [12.9]*	*11.4 ± 7.6 [11.8]*	*H = 0.04; P = .844*
**Amp (µV)**	*358 ± 433*	*364 ± 488*	*H = 0.86; P = .354*
**Lat0 (ms)**	*24.9 ± 2.1*	*24.8 ± 1.5*	*H = 0.01; P = .921*
**Lat1 (ms)**	*29.6 ± 2.2*	*28.6 ± 1.4*	*H = 3.00; P = .084*
**Lat2 (ms)**	*33.8 ± 2.4*	*32.9 ± 1.6*	*H = 2.67; P = .103*
**Lat1-0 (ms)**	*4.7 ± 1.4*	*3.8 ± 0.6*	*H = 5.80; P = .016*
**Lat2-1 (ms)**	*4.2 ± 1.0*	*4.2± 1.1*	*H = 0.08; P = .778*
**ERSP_all_**	*29.6 ± 6.6*	*22.3 ± 6.1*	*H = 9.73P = .002*
**ERSP_HS_**	*34.9 ± 7.8*	*26.8 ± 6.0*	*H = 10.08; P = .001*
**ERSP_NHS_**	*24.9 ± 7.1*	*16.9 ± 5.6*	*H = 10.81; P = .001*
**ERSP_adj_**	*28.2 ± 9.4*	*19.9 ± 5.1*	*H = 9.85; P = .002*

Data are shown as mean ± SD.

Abbreviations: Amp, peak-to-peak MEP amplitude; dist2tumor, closest distance between stimulation site (SS) and tumor border; distHS2tum, closest distance between the hotspot and the tumor border; EF_HS_, strength of the induced electric field at the functional hotspot; ERSP, event-related spectral perturbation for 20 ≤ *t* ≤ 40 ms and 37 ≤ *f* ≤ 200 Hz; ERSP_adj_, for SS with dist2tum ≤15 mm; ERSP_all_, averaged across all SS; ERSP_dist_, for with dist2tum >15 mm; ERSP_HS_, for SS with dist2HS ≤ 10 mm; ERSP_NHS_, for SS with dist2HS >10 mm; IQR, interquartile range; Lat0, MEP onset latency; Lat1, latency of the strongest positive peak; Lat1-0, difference between Lat1 and Lat0; Lat2, latency of the strongest negative peak; Lat2-1, difference between Lat2 and Lat1; MEP+, TMS trials with MEP peak-to-peak amplitudes of >20 µV; RMT, resting motor threshold.

The resulting MEP showed a high intersubject variability (Figure 1E). However, conventional TMS parameters did not differ between *IDH-mt* and *IDH-wt* tumors (Table 2). The MEP were transferred to the frequency domain showing a high spectral synchronization (ie, positive ERSP) for frequencies up to 500 Hz and a time period of 20 to 40 ms after the TMS (Figure 1E).

**Figure 2.**
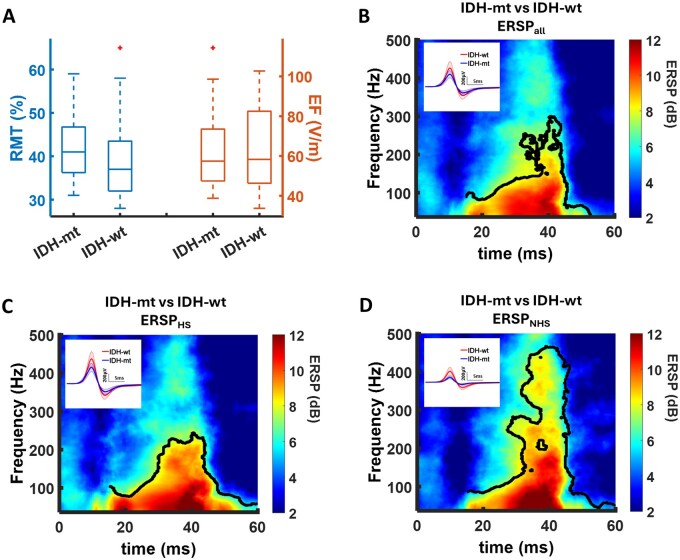
Differences in corticospinal output gain between *IDH-mt* and *IDH-wt* patients. (A) There were no significant group differences in the resting motor threshold (RMT) between *IDH-mt* and *IDH-wt* patients. There was no significant group difference in the strength of the electric field (EF) induced by the magnetic pulse. (B) Cluster-based permutation analysis was applied to evaluate group differences in the time-frequency representation of motor-evoked potentials of all stimulation sites (ERSP_all_). There was a significant increase in the event-related spectral perturbation (ERSP for 37 ≤ *f* ≤ 200 Hz and 20-40 ms after TMS), in *IDH-mt* compared to *IDH-wt* patients. A similar hyperexcitability was observed for MEPs at functional hotspot (ERSP_HS_) (C) as well as beyond the functional hotspot (ERSP_NHS_) (D). Significant time-frequency bins are outlined in black (*P* < .05, cluster corrected). The inlays demonstrate the corresponding MEP time-series.

The conventional electrophysiological indicator of cortical excitability (ie, RMT) did not differ between the *IDH* groups (Figure 2A, Table 2). Notably, the RMT evaluates cortical excitability at the functional hotspot, exclusively. Multivariate linear regression of RMT values, *R*^2^ = 0.204, *F*_(3,35)_ = 2.990, *P* = .044, in relation to the *IDH* status, tumor *GRADE*, and *AED* intake identifies AED intake as the only significant predictor (β = −0.421, *T* = −2.625, *P* = .013). In contrast, the EF strength induced by TMS to the hotspot was not affected by these factors, *R*^2^ = 0.014, *F*_(3,35)_ = 0.162, *P* = .921.

A cluster-based permutation test (correcting for multiple comparisons) was performed to evaluate significant group differences in the time-frequency representations of MEPs. Overall, we observed an increased ERSP in *IDH-mt* patients when averaging across all stimulation sites (ERSP_all_, Figure 2B, Table 2), indicating an enhanced cortical excitability. This augmented corticospinal output gain was detected for both the functional hotspot (ERSP_HS_, Figure 2C, Table 2) and the surrounding motor-eloquent brain tissue (ERSP_NHS_, Figure 2D, Table 2). In contrast, neither AED intake (AED+ vs AED-) nor the histological tumor grading (HGG vs LGG) affected the corticospinal output gain in the vicinity of the tumors (Supplementary Figure S1). A multivariate linear regression controlling for tumor *GRADE*, *AED* intake, and *EF_HS_* confirmed *IDH* status as the main predictor of ERSP_all_, *R*^2^ = 0.293, *F*_(4,34)_ = 3.518, *P* = .017; ERSP_HS_, *R*^2^ = 0.343, *F*_(4,34)_ = 4.437, *P* = .005; and ERSP_NHS_, *R*^2^ = 0.343, *F*_(4,34)_ = 4.300, *P* = .007 (Table 3).

**Table 3.** Results of the multivariate linear regression predicting the event-related spectral perturbation (ERSP) depending on the IDH status (*IDH-mt* vs *IDH-wt*), intake of antiepileptic drugs (AED), tumor GRADE (LGG vs HGG) and the strength of the electric field at the hotspot (EF_HS_)

	*ERSP_all_*				*ERSP_HS_*				*ERSP_NHS_*				*ERSP_BTI_*			
	*B*	*β*	*T*	*P*	*B*	*β*	*T*	*P*	*B*	*β*	*T*	*P*	*B*	*β*	*T*	*P*
**IDH**	9.639	0.553	3.652	<.001	10.277	0.602	4.126	<.001	10.366	0.578	3.927	<.001	12.885	0.553	3.456	.002
**AED**	−0.214	−0.011	−0.072	.943	2.051	0.108	0.728	.471	−2.580	−0.131	−0.870	.391	−1.105	−0.044	−0.266	.792
**GRADE**	−0.993	−0.055	−0.352	.727	−0.483	−0.028	−0.181	.857	−2.801	−0.153	−0.995	.327	−1.731	−0.073	−0.438	.664
**EF_HS_**	−0.011	−0.026	−0.179	.859	0.001	0.004	0.025	.980	0.012	0.028	0.194	.847	−0.013	−0.023	−0.147	.884

Abbreviations: B, unstandardized regression coefficient; *dist2HS*, distance between stimulation site (SS) and the functional hotspot (HS); *dist2tum*, distance between SS and the tumor border; *ERSP_all_*, averaged across all SS; *ERSP_BTI_*, averaged ERSP for SS with dist2HS >10 mm and 11 mm ≤ dist2tum ≤15 mm); *ERSP_HS_*, averaged ERSP for SS with dist2HS ≤10 mm; *ERSP_NHS_*, averaged ERSP for SS with dist2HS >10 mm; *P*, *P*-value; *T*, *t*-statistics; β, standardized regression coefficient.

**Figure 3.**
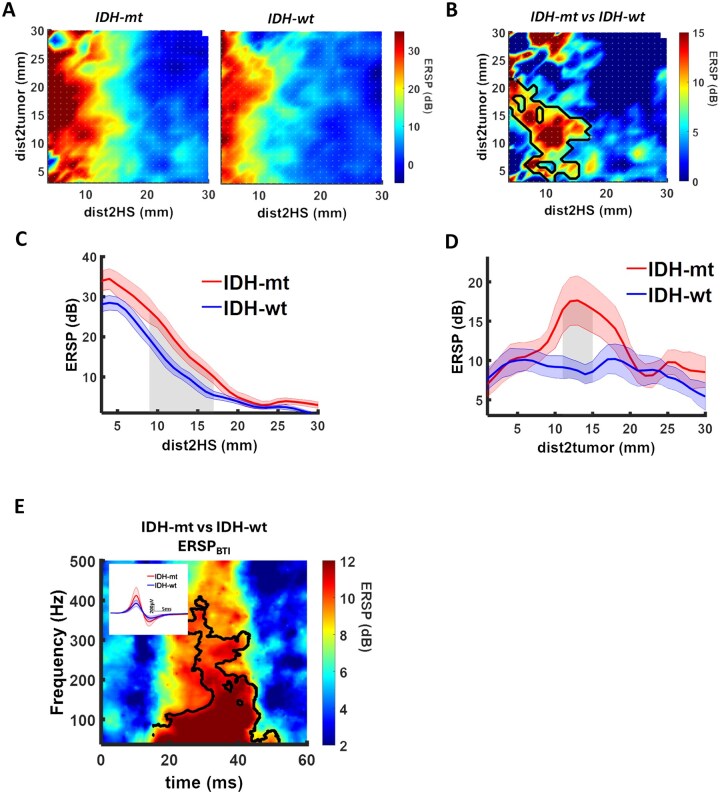
Peritumor hyperexcitability of *IDH-mt* glioma. To explore the spatial relationship between the corticospinal output gain, the tumor and the functional hotspot we rearranged stimulation sites (white dots) according to dist2HS (*x*-axis) and dist2tum (*y*-axis) in a 2-dimensional coordinate system (ie, spatial map). ERSP values represent the mean ERSP amplitude within the significant TF cluster (37-200 Hz, 20-40 ms). ERSP_BTI_ refers to the mean ERSP averaged across stimulation sites located 11-15 mm from the tumor border and more than 10 mm from the functional hotspot, defining the operational brain-tumor interface (BTI) zone. *IDH-mt* and *IDH-wt* patients showed a similar spatial ERSP distribution with higher excitability close to the functional hotspot (A). (B) Cluster-based permutation analysis identified group differences in the adjacent matrices with an increase of excitability in IDH-mt patients. Significant bins are outlined in black (*P* < .05, cluster corrected). Averaging the spatial maps along the *y*-axis (dist2tumor) implies a linear decay of ERSP as the dist2HS increases with an increased excitability for *IDH-mt* patients (C). In contrast, averaging the spatial maps along the *x*-axis (dist2HS) indicates a significant increase of ERSP in *IDH-mt* patients adjacent to the tumor (D). Cluster-based permutation test reaffirmed an *ERSP_BTI_* increase in *IDH-mt* patients. Significant time-frequency bins are outlined in black (*P* < .05, cluster corrected). The inlays demonstrate the corresponding MEP time-series (E).

To evaluate the exact spatial relationship between ERSP, *dist2tum* and *dist2HS*, we averaged the spatial maps across *IDH-mt* and *IDH-wt* patients, respectively. In both groups, maximum ERSP was evoked within 10 mm to the hot spot (Figure 3A). A cluster-based permutation test was performed to evaluate significant group differences in the spatial maps. This analysis indicated an increase of ERSP at the BTI of *IDH-mt* glioma (Figure 3B). Notably, this ERSP increase was not attributed to fluctuations of the stimulation intensity *EF* (Supplementary Figure S1). Averaging the spatial maps along the *y*-axis (dist2tumor) implies a linear decay of ERSP as the dist2HS increases with an increased excitability for *IDH-mt* patients (Figure 3C). In contrast, averaging the spatial maps along the *x*-axis (dist2HS) indicates a significant increase of ERSP in *IDH-mt* patients adjacent to the tumor (Figure 3D). Cluster-based permutation test reaffirmed an *ERSP_BTI_* increase in *IDH-mt* patients (Figure 3E, Table 2). Finally, a multivariate linear regression controlling for tumor *GRADE*, *AED* intake, and *EF_HS_*, *R*^2^ = 0.293, *F*_(4,34)_ = 3.529, *P* = .016, confirmed *IDH* status as the main predictor of ERSP_BTI_ (Table 3).

### Influence of Other Histomolecular Characteristics

For high-grade glioma, we repeated the multivariate regression analysis on ERSP_all_, *R*^2^ = 0.423, *F*_(5,18)_ = 2.636, *P* = .059; ERPS_HS_, *R*^2^ = 0.550, *F*_(5,18)_ = 4.407, *P* = .009; ERSP_NHS_, *R*^2^ = 0.550, *F*_(5,18)_ = 4.150, *P* = .012; and ERSP_BTI_, *R*^2^ = 0.494, F_(5,18)_ = 2.926, *P* = .049, to evaluate the effect of the IDH status while controlling for other histomolecular characteristics, such as *ATRX* and *MIB* expression, the MGMT status, and microscopic anaplasia signs. The *IDH* status was the only significant predictor for ERSP_all_, *B* = 11.855, β = 0.644, *T* = 1.959, *P* = .066; ERPS_HS_, *B* = 13.990, β = 0.739, *T* = 2.549, *P* = .020; ERSP_NHS_, *B* = 17.727, β = 1.011, *T* = 3.383, *P* = 0.004; and ERSP_BTI_, *B* = 24.238, β = 1.028, *T* = 3.105, *P* = .007.

In IDH-mutant gliomas, ERSP_all_ did not differ significantly between LOH+ (oligodendroglioma) and LOH− (astrocytoma) tumors (30.3 ± 11.0 dB, 95% confidence interval [CI] = 23.3-39.5 and 30.7 ± 6.7 dB, 95% CI = 27.1-34.6), H(1) = 0.26, *P* = .612 (Supplementary Figure S1). Given the small number of LOH− cases (n = 7), this analysis is likely underpowered. In line, multivariate regression analysis on ERSP_all_, *R*^2^ = 0.086, *F*_(3,15)_ = 0.473, *P* = .706; ERPS_HS_, *R*^2^ = 0.069, *F*_(3,15)_ = 0.368, *P* = .777; ERSP_NHS_, *R*^2^ = 0.106, *F*_(3,15)_ = 0.592, *P* = .630; and ERSP_BTI_, *R*^2^ = 0.107, *F*_(3,15)_ = 0.521, *P* = .676,did not depict any significant effect of the LOH status, *ATRX*, or *MIB* expression.

## Discussion

The aim of the study was to provide in vivo evidence that the *IDH* status of a glioma affects cortical excitability. The present data demonstrate an increased corticospinal output of the peritumoral cortex in *IDH-mt* compared with *IDH-wt* gliomas. The AED intake, tumor grading, and other histological/molecular characteristics did not affect cortical excitability. This hyperexcitability might contribute to epileptogenesis in *IDH-mt* patients. However, the pathophysiology of epileptogenesis in *IDH-mt* gliomas remains unclear.

It was not until recently that seminal studies have proven a functional integration of gliomas into the peritumoral neural network.^2^^,^^3^^,^^7^ The *in vitro* data suggest a bidirectional glioma-to-neuron communication via glutamatergic neurogliomal synapses at tumor microtubes (TM). Here, activation of glutamatergic neurogliomal synapses drive tumor cell growth and migration by depolarization-induced calcium-ion influx.^2^^,^^3^^,^^7^ At the same time, gliomas have been shown to increase neuronal activity in the peritumoral tissue.^2^ These signals are inhibited by pharmacological and genetic blockage of the glutamate synaptic transmission.^2^^,^^7^ In *IDH-mt* gliomas, however, TM and glutamatergic synapsis are rare or absent ^3^^,^^7^ although epileptogenesis is more common in these gliomas than in other glioma subtypes.^33^

In contrast, recent in vitro study suggests that *IDH-mt* gliomas increase neuronal excitability in the surrounding tissue by metabolic interaction.^4^ This is consistent with the observation that peritumoral excitability is normalized following tumor resection ^5^ and that *IDH* inhibitors (eg, ivosidenib) can improve seizures in *IDH*-mt glioma patients.^34^ In vivo evidence for modulation of cortical excitability at the BTI of *IDH*-mt glioma, however, is spare.^2^^,^^3^ One of the few in vivo human studies, Venkatesh et al, provided indirect evidence for peritumoral hyperexcitability in 3 *IDH-wt* glioma patients demonstrating an increased power of gamma frequencies in intraoperative electrocorticographic recordings. As gamma oscillations correlate with neuronal firing rate, they concluded a cortical hyperexcitability.^2^

Transcranial magnetic stimulation is a widely used tool to noninvasively probe cortical excitability in vivo.^13^^,^^14^ For example, an impaired intracortical inhibition has been shown to affect cortico-spinal output after TMS (ie, RMT and MEP morphology)^35^^,^^36^ and to attribute to epileptogenesis^36^ in idiopathic generalized epilepsy. This cortical excitability is reversed after GABAergic AED intake.^17^^,^^18^^,^^37^ In contrast, most TMS studies fail to demonstrate significant RMT and MEPs changes due to a high intersubject variability in glioma patients.^19–21^^,^^38–40^ No study, however, has considered the spatial relationship of the stimulation sites to the tumor and functional hotpot, which affects corticospinal output after TMS^19–21^^,^^38–40^ and the IDH status.^19–22^ In the present study, we have demonstrated an increased cortico-spinal output in the vicinity of *IDH-mt* glioma compared with *IDH-wt* glioma. There was no increased cortical excitability per se (ie, at the functional hotspot). Maximal cortical excitability was observed approximately 10 mm beyond the radiological tumor margin. The BTI is therefore not strictly defined by the MRI border, but functionally broader, and our findings reflect alterations within this extended peritumoral field. This interpretation is consistent with previous structural, metabolic, and electrophysiological evidence demonstrating that gliomas induce a “functional penumbra” in the surrounding cortex that extends beyond the radiologically visible lesion.^2^^,^^4^^,^^8^^,^^41^ Our study is observational and therefore cannot identify the mechanisms underlying the increased peritumoral excitability in IDH-mutant gliomas. Differences in growth dynamics and infiltration patterns between IDH-mutant and IDH-wildtype tumors may contribute to distinct forms of network reorganization in the surrounding cortex.^8^^,^^41^ In addition, IDH mutations lead to characteristic metabolic alterations, including D-2-hydroxyglutarate accumulation and downstream mTOR hyperactivation, both of which have been linked to increased neuronal excitability in preclinical models.^4^ Finally, recent work shows that neuronal activity can modulate glioma progression through synaptic and activity-dependent signaling.^2^^,^^3^ Although our data cannot test these mechanisms directly, they provide an in vivo functional phenotype that is compatible with these emerging concepts. Further understanding of the mechanisms at the BTI might pave the way for new approaches to glioma therapy based on modulation of the synaptic neuron-to-glioma communication, for example, by repetitive TMS.^9^ Additionally, this knowledge might affect surgical strategy. For example, supramarginal resections of gliomas are associated with better seizure outcome.^42^^,^^43^

Finally, some methodological aspects should be mentioned. Negative subgroup findings such as the comparison between LOH+ (oligodendroglioma) and LOH− (astrocytoma) within the IDH-mutant cohort should be interpreted with caution. These analyses are limited by small subgroup sizes, meaning that moderate effects may remain undetected. Thus, the absence of a statistically significant difference does not exclude the possibility that a true association exists and might be detected in larger cohorts. Methodological factors may have affected the electrophysiological evaluation of the BTI. For example, spatial resolution of navigated TMS in peritumoral cortex is limited (approximately 5-10 mm) and influenced by cortical geometry. The triangulation-based interpolation required to generate continuous spatial maps smooths the data and may shift local maxima by several millimeters, especially in areas with sparse sampling near the tumor border. It is well known that conventional TMS parameters (ie, MEP latencies and peak-to-peak amplitudes, RMT) exerts a high intersubject and intrasubject variability impeding interpretation.^13^^,^^19^^,^^20^^,^^30^^,^^44^^,^^45^ Innovative time-frequency analysis provides an alternative technique for describing MEP characterized by a higher reliability than conventional time-series parameters.^26^^,^^27^^,^^30^ In the frequency domain, MEP are represented by a high synchronization (ERSP) of a wide range of frequency, with a local maximum between 100 and 250 Hz.^26^^,^^27^^, ^^30^ Recent studies have shown that ERSP changes in this frequency band enable the detection of fine intrasubject and intersubject differences in glioma patients that would have been missed by convention techniques.^26^^,^^27^^,^^30^ Hence, hitherto studies might have failed to detect MEP differences in glioma subtypes.

## Conclusion

The present study shows that *IDH-mt* gliomas affect the brain’s response to an external magnetic input beyond tumor borders. This peritumoral hyperexcitability could contribute to epileptogenesis in this glioma subtype. As interactions at the BTI are known to also affect tumor growth,^7^ understanding the mechanisms paves the way for new therapeutic approaches in gliomas.^9^

## Supplementary Material

vdag071_Supplementary_Data

## Data Availability

The data that support the findings of this study are available from the corresponding author upon reasonable request.
