# Supplementary material for: Increased cortical excitability to transcranial magnetic stimulation at the brain-tumor interface of IDH1-mutant gliomas
Source: Neurooncol Adv. 2026 Mar 15;8(1):vdag071. doi: 10.1093/noajnl/vdag071 (PMC13035068; doi:10.1093/noajnl/vdag071)
Supplement: vdag071_Supplementary_Data [file vdag071_supplementary_data.zip › Supplementary Figure Legend.docx]

**Suppl. figure 1:** (***A***) There were no significant group differences in the time-frequency representation of MEP in patients with and without antiepileptic drug intake (AED+ vs AED). ***(B***) There were no significant group differences in the time-frequency representation of MEP in patients with high- and low-grade gliomas (HGG vs LGG). (***C***) There were no significant group differences in the electric field strength (EF) applied in *IDH-mt* and *IDH-wt* patients that could have affected the corticospinal output. (D) In *IDH-mt* glioma, LOH+ (i.e., oligodendroglioma) did not show any difference in peritumoral excitability when compared to LOH-.
