# Supplementary figures and images for: Increased cortical excitability to transcranial magnetic stimulation at the brain-tumor interface of IDH1-mutant gliomas
Source: Neurooncol Adv. 2026 Mar 15;8(1):vdag071. doi: 10.1093/noajnl/vdag071 (PMC13035068; doi:10.1093/noajnl/vdag071)

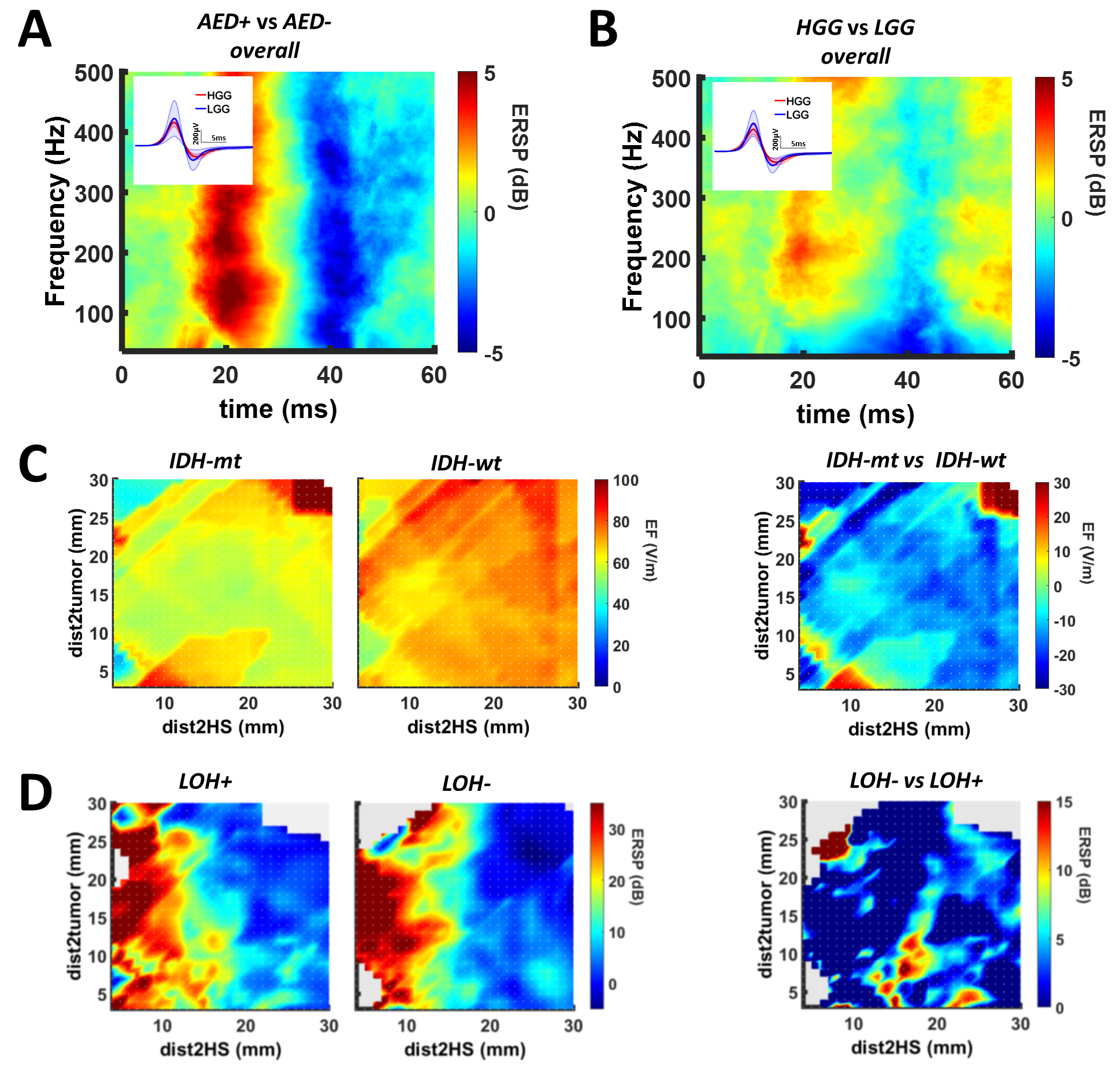

Supplement: vdag071_Supplementary_Data [file vdag071_supplementary_data.zip › Figure S1.tiff]
